# FDA-Approved HIV-1 Capsid Inhibition With Lenacapavir: A Paradigm Shift in Pre-exposure Prophylaxis

**DOI:** 10.7759/cureus.92043

**Published:** 2025-09-11

**Authors:** Kashaf Sanaullah, Abdul Haseeb Hasan, Muhammad Ali Abid

**Affiliations:** 1 Medicine, King Edward Medical University, Lahore, PAK

**Keywords:** cabotegravir, capsid inhibitor, fda, hiv, hiv-1 capsid, human immunodeficiency virus, lenacapavir, purpose trial, who, yeztugo

## Abstract

Human immunodeficiency virus type 1 (HIV-1) remains a major global health challenge, with over 40 million people currently living with the infection. While daily oral antiretroviral therapy and pre-exposure prophylaxis (PrEP) are highly effective, adherence barriers limit their impact. Lenacapavir, a first-in-class HIV-1 capsid inhibitor with a unique multistage mechanism of action, offers potent antiviral activity and sustained plasma concentrations that enable twice-yearly subcutaneous dosing. In two phase 3 randomized controlled trials, lenacapavir demonstrated superior efficacy to oral PrEP regimens, with near-complete prevention of HIV acquisition across diverse populations, including cisgender women, men, and gender-diverse individuals. The safety profile was acceptable, though injection site reactions were common, and the drug’s prolonged pharmacokinetic tail necessitates strict HIV testing before each dose to prevent resistance. Implementation challenges include programmatic training, drug-drug interaction considerations, and disparities in PrEP uptake, particularly among women, Black and Hispanic/Latino populations, and individuals in the US South. While currently approved for PrEP only in the US, broader global access will depend on regulatory decisions, WHO guidance, and procurement strategies. Lenacapavir represents a significant advance in HIV prevention, with the potential to expand protection options and reduce new infections if integrated into equitable and robust public health programs.

## Editorial

Introduction

For more than four decades, the pandemic of human immunodeficiency virus type 1 (HIV-1) has remained a serious threat to public health. It has claimed over 42 million lives, and about 40 million people are living with HIV worldwide [1]. In 2023, the US saw over 100 new HIV diagnoses per day [2]. Advances in highly active combination antiretroviral therapy have transformed HIV from a frequently fatal disease into a manageable chronic condition. Most people with HIV can now achieve near-normal life expectancy with modern treatments. The current standard of once daily oral regimens is safe and highly effective. Despite this success, the requirement for lifelong near-perfect adherence remains a significant barrier. Suboptimal adherence can lead to treatment failure and the development of drug resistance [1].

There is, therefore, a significant unmet medical need for antiretroviral agents that allow less frequent dosing and reduce the burden of daily therapy. Long-acting options can improve convenience and adherence, and can lessen the emotional burden and stigma that some people experience with daily pills [1]. In addition, there is a clear need for effective pre-exposure prophylaxis (PrEP) to prevent infection before it occurs and to curb transmission at the population level. PrEP can protect individuals at risk and complement treatment efforts to reduce new infections. Currently, the World Health Organization (WHO) suggests oral PrEP, the dapivirine vaginal ring, and long-acting injectable cabotegravir (CAB-LA) for HIV PrEP [3].

HIV-1 capsid

The HIV-1 capsid protein forms the core lattice and is essential for proper capsid assembly, stability, and intracellular trafficking through the cytoplasm and nuclear pore into the nucleus. [1]. Its multiple critical roles and high genetic fragility make the capsid an attractive target for therapeutic interventions like lenacapavir [1].

Lenacapavir

Lenacapavir is a first-in-class HIV-1 capsid inhibitor with a multistage mechanism of action that distinguishes it from other antiretroviral classes [1,2]. It binds at the interface between adjacent capsid subunits and exerts potent antiviral activity at both early and late stages of the viral life cycle [1]. During early infection, it hyper-stabilizes mature capsid cores and impairs nuclear import, and during late stages, it disrupts proper virion maturation, producing noninfectious particles [1]. The lenacapavir binding site overlaps with host cofactors required for nuclear trafficking, and lenacapavir’s engagement of this pocket therefore also interferes with capsid-host interactions that are important for productive infection [1]. Because of its unique mechanism of action, lenacapavir effectively inhibits both wild-type HIV-1 and variations that are resistant to other kinds of antiretroviral drugs [1]. Lenacapavir’s picomolar potency, combined with low systemic clearance and slow-release kinetics, enables multiple dosing modalities, including a twice-yearly injectable formulation [1].

FDA approval and dosing

The US Food and Drug Administration (FDA) has approved injectable lenacapavir (Yeztugo, Gilead Sciences, Foster City, CA) for PrEP to reduce the risk of sexually acquired HIV-1 in adults and adolescents weighing at least 35 kg (approximately 77 lbs), making it the first twice-yearly PrEP option in the US [2,3]. The approved injection formulation is 463.5 mg per 1.5 mL [2]. The recommended initiation comprises day 1 subcutaneous injections (two 1.5 mL injections totaling 927 mg) combined with 600 mg orally (two 300 mg tablets), followed by a day 2 oral 600 mg dose, with continuation injections of 927 mg subcutaneously every 26 weeks (±2 weeks). If a scheduled six-month injection is delayed by more than two weeks, interim oral tablets (300 mg once weekly) may be taken for up to six months until injections resume. Restart with initiation dosing if clinically acceptable, if more than 28 weeks have passed since the last injection, and Yeztugo tablets have not been used [2].

PURPOSE 1 trial

PURPOSE 1 was a phase 3, double-blind, randomized controlled trial involving adolescent girls and young women in South Africa and Uganda [4]. The trial enrolled 5,338 participants who were initially HIV negative and were assigned in a 2:2:1 ratio to receive either subcutaneous lenacapavir every 26 weeks, daily oral emtricitabine-tenofovir alafenamide (F/TAF), or daily oral emtricitabine-tenofovir disoproxil fumarate (F/TDF), and all participants also received the alternate subcutaneous or oral placebo [4]. Participants were scheduled for follow-up visits at weeks 4, 8, and 13, and subsequently at 13-week intervals. The analyses were conducted after 52 weeks of follow-up. Overall, 55 incident HIV infections were observed, with zero infections among 2,134 participants in the lenacapavir group corresponding to 0 per 100 person-years (95% confidence interval (CI): 0.00-0.19), 39 infections among 2,136 participants in the F/TAF group corresponding to 2.02 per 100 person-years (95% CI: 1.44-2.76), and 16 infections among 1,068 participants in the F/TDF group corresponding to 1.69 per 100 person-years (95% CI: 0.96-2.74) [4]. Background HIV incidence in the screened population was 2.41 per 100 person-years (95% CI: 1.82-3.19). HIV incidence with lenacapavir was significantly lower than background HIV incidence with an incidence rate ratio of 0.00 (95% CI: 0.00-0.04; P < 0.001), and was also considerably lower than HIV incidence with F/TDF with an incidence rate ratio of 0.00 (95% CI: 0.00-0.10; P < 0.001) [4]. HIV incidence with F/TAF did not differ significantly from background incidence, with an incidence rate ratio of 0.84 (95% CI: 0.55-1.28; P = 0.21), and no evidence of a meaningful difference in HIV incidence was observed between F/TAF and F/TDF, with an incidence rate ratio of 1.20 (95% CI: 0.67-2.14). Adherence to both F/TAF and F/TDF was low. No new safety concerns were identified. Injection site reactions were more common in the lenacapavir group at 68.8% compared with 34.9% in the placebo injection groups (F/TAF and F/TDF combined), and four participants in the lenacapavir group, representing 0.2%, discontinued the trial regimen owing to injection site reactions. Overall, no participants receiving twice-yearly lenacapavir acquired HIV infection, and HIV incidence with lenacapavir was significantly lower than both background incidence and incidence with F/TDF [4].

**Table 1:** Characteristics of PUROSE 1 trial

	Lenacapavir	F/TAF	F/TDF
Number of participants	2134	2136	1068
Median age (range)	21 (16-25)	21 (16-26)	21 (16-25)
Number of new HIV infections	0	39	16
Incidence ratio of new HIV infections	0	0.84	1.20
Injection-site reaction leading to premature discontinuation of the trial regimen	4	0	0

PURPOSE 2 trial

PURPOSE 2 was a phase 3, double-blind, randomized controlled trial involving cisgender men or transgender women in the US, Brazil, Thailand, South Africa, Peru, Argentina, and Mexico [5]. The trial enrolled 3,265 participants, and they were randomly assigned in a two-to-one ratio to receive either subcutaneous lenacapavir every 26 weeks or daily oral emtricitabine-tenofovir disoproxil fumarate [5]. Participants were scheduled for follow-up visits at weeks 4, 8, and 13, and subsequently at 13-week intervals. The analyses were conducted after 52 weeks of follow-up. Overall, 11 incident HIV infections were observed, with two infections in the lenacapavir group corresponding to 0.10 per 100 person-years (95% CI: 0.01-0.37) and nine infections in the F/TDF group corresponding to 0.93 per 100 person-years (95% CI: 0.43-1.77) [5]. Background HIV incidence in the screened population was 2.37 per 100 person-years (95% CI: 1.65-3.42) [5]. HIV incidence in the lenacapavir group was significantly lower than the background incidence with an incidence rate ratio of 0.04 (95% CI: 0.01-0.18; P < 0.001) and was considerably lower than incidence in the F/TDF group with an incidence rate ratio of 0.11 (95% CI: 0.02-0.51; P = 0.002). No safety concerns were identified [5]. A total of 26 of 2,183 participants, or 1.2% in the lenacapavir group, and 3 of 1,088 participants, or 0.3% in the F/TDF group, discontinued the trial regimen because of injection site reactions [5]. Overall, twice-yearly lenacapavir was associated with significantly lower HIV incidence than both the background incidence and the incidence observed with daily oral F/TDF [5].

**Table 2:** Characteristics of the PURPOSE 2 trial

	Lenacapavir	F/TDF
Number of participants	2183	1088
Median age (range)	28 (17-74)	29 (17-73)
Number of new HIV infections	2	9
Incidence ratio of new HIV infections	0.04	0.11
Injection-site reaction leading to premature discontinuation of the trial regimen	26 (1.2%)	3 (0.3%)

Limitations

Clinical and programmatic limitations are essential to recognize. Use of Yeztugo requires strict HIV screening before initiation and before each injection because the long-acting nature of the drug and prolonged residual concentrations can select for resistance if a person with undiagnosed or subsequently acquired HIV receives effective monotherapy [2]. Residual systemic concentrations may persist for up to 12 months or longer after the last injection, and this has implications for resistance risk and for the timing of alternative prevention strategies after discontinuation [2]. Common adverse events observed in trials included injection-site reactions, headache, and nausea [2]. Injection site reactions were common in trials, and if injections are given improperly into the dermis rather than subcutaneously, serious local injuries, including necrosis and ulceration, have been reported [2,4,5]. Correct subcutaneous administration and programmatic training are therefore essential. Pharmacologic interactions with strong or moderate CYP3A inducers can reduce lenacapavir concentrations and require dosage modification, and coadministration with certain combinations of inhibitors is not recommended [2]. PrEP must also be accessible and taken up by people at risk. CDC data show that in 2022, only about one-third of US individuals who met PrEP criteria were prescribed it [2]. Uptake also remains inadequate to stop transmission at the population level, and significant gaps persist for women, Black and Hispanic or Latino people, and for people in the US South. Key barriers include adherence challenges, stigma, and low awareness of PrEP among clinicians and patients [2]. Regulatory approvals for lenacapavir for PrEP are currently limited to the US, and broader global availability depends on national regulatory decisions, WHO guidance, and procurement mechanisms [2,3]. These factors together create practical constraints on rollout and underscore the need for careful screening, counseling, and systems to ensure correct dosing and follow-up.

Future directions

The introduction of lenacapavir as the first twice-yearly injectable for HIV PrEP in the US marks a significant advancement in HIV prevention strategies [2]. Future research should focus on expanding the evidence base across diverse populations, including women, transgender individuals, people who inject drugs, and populations in regions with high HIV incidence but limited access to prevention services. Ongoing and planned clinical trials in varied epidemiologic settings will be critical to determine real-world effectiveness, safety, and acceptability, particularly in low- and middle-income countries where the HIV burden is highest.

Equally important will be the evaluation of implementation models that integrate lenacapavir into existing health systems, ensuring that HIV screening, counseling, and injection delivery are conducted safely and consistently. Strategies to minimize missed doses and address the “long tail” pharmacokinetic period will require programmatic protocols, clinician education, and patient engagement.

From a regulatory perspective, the expansion of approvals beyond the US will depend on timely submissions to national authorities, inclusion in WHO guidelines, and global procurement mechanisms. Cost-effectiveness analyses, pricing negotiations, and licensing agreements will play pivotal roles in determining equitable access. Furthermore, health communication campaigns and clinician training should address stigma, raise awareness of long-acting PrEP options, and ensure informed choice between oral, injectable, and other emerging modalities.

In the longer term, combination long-acting products that incorporate multiple antiretroviral agents or couple HIV prevention with contraception may further enhance prevention efficacy and convenience. The experience with lenacapavir will also pave the way to the development of future capsid inhibitors and other long-acting agents, contributing to a broader paradigm shift toward less frequent dosing in both HIV prevention and treatment. By addressing scientific, regulatory, and programmatic challenges in parallel, lenacapavir’s promise as a transformative tool in ending the HIV epidemic can be fully realized.

Conclusion

Lenacapavir represents a significant advance in HIV prevention as a twice-yearly injectable PrEP. The trials support high preventive efficacy with an acceptable safety profile. However, injection site reactions and the prolonged pharmacokinetic tail raise concerns about resistance that require strict HIV testing and correct subcutaneous administration. The real-world benefit of this approach will depend on equitable access, uptake by populations at risk, and robust programmatic systems for screening, counseling, and follow-up. Continued clinical evaluation and implementation research are needed to confirm effectiveness across diverse populations and to inform broader regulatory and programmatic adoption.
